# Life expectancy and survival analysis of patients with diabetes compared to the non diabetic population in Bulgaria

**DOI:** 10.1371/journal.pone.0232815

**Published:** 2020-05-11

**Authors:** Konstantin Tachkov, Konstantin Mitov, Yordanka Koleva, Zornitsa Mitkova, Maria Kamusheva, Maria Dimitrova, Valentina Petkova, Alexandra Savova, Miglena Doneva, Dimitar Tcarukciev, Vasil Valov, Galia Angelova, Manoela Manova, Guenka Petrova

**Affiliations:** 1 Faculty of Pharmacy, Medical University of Sofia, Sofia, Bulgaria; 2 Tulane University, New Orleans, Louisianna, United States of America; 3 University endocrinology hospital, Medical University of Sofia, „Ivan Penchev“, Sofia, Bulgaria; 4 NovoNordisk Pharma, Sofia, Bulgaria; 5 Institute of informatics and informational technologies, Bulgarian academy of sciences, Sofia, Bulgaria; Jawaharlal Nehru University, INDIA

## Abstract

**Aims:**

To evaluate the expected life expectancy in patients with diabetes in Bulgaria and to compare it to the expected life expectancy of the non-diabetic population in the country.

**Methods:**

It is a retrospective observational population study on individuals diagnosed with diabetes, compared to the non-diabetic population in Bulgaria for the period 2012–2015. Data from the national diabetes register and national statistical institute were used to construct life-tables with probability of survival with t-test and Chi Square test. Confounder analysis was done by age, sex, and type of diabetes. All-cause mortality and deaths in diabetic patients were analyzed. Kaplan-Meier survival curves were constructed for each age group and a log-rank analysis was conducted.

**Results:**

Average life expectancy in the non-diabetic population, patients with Type 1 DM and with Type 2 DM is 74.8; 70.96 and 75.19 years, respectively. For 2012–2015 the mortality in the non-diabetic population remained constant and lower (average—1.48%) compared to type-1 DM (5.25%) and Type-2 (4.27%). Relative risk of death in diabetics was higher overall (12%), after the age of 70 before which the relative risk was higher for the non-diabetic population. This was observed as a trend in all analyzed years.

**Conclusion:**

Patients with type 2 DM have a longer life-expectancy than patients with type-1 DM and overall Diabetics life expectancy equals that of the non-diabetic population, which could suggest improved disease control and its associated complications in Bulgaria. Male diabetics show slightly longer life expectancy than their counterparts in the non-diabetic population, by a marginal gain of 0.6 years for the entire observed period. Life expectancy in diabetic women increased by 1.3 years, which was not observed in the non-diabetic population. Prevalence of diabetes was higher for women. Improved diabetes control may explain this gain in life; however other studies are needed to confirm this.

## Introduction

Diabetes is still one of the major health care concerns worldwide among the aging population– 422 million adults were living with diabetes in 2014. Data of the World Health Organization (WHO) show that the prevalence of diabetes, especially diabetes type 2 has been steadily increasing in the past three decades, mainly in the low- and middle-income countries. Diabetes-related micro- and macro vascular complications increase the overall risk of premature death. [1] One and a half million deaths due to diabetes were registered in 2012, as almost half of them occurred before the age of 70 years. [2] Adherence to therapy and development of different disease management programmes, however, lead to better control of the risk factors, reduce diabetes-related complications and increase survival. [3], [4] In this respect, estimation of life expectancy (LE) in patients with diabetes compared to the non-diabetic population is an important tool to evaluate the impact of diabetes on the population from one side, and to assess the modifiable risk factors from other side. [3]. Most of the studies estimating life expectancy among patients with diabetes show that life expectancy decreases with age, and is shorter than LE among the general population [5], [6]. Additionally, an increase in the time spent in morbidity is observed (i.e. longer duration of disease). [7]

In Bulgaria current prevalence estimates put the number of people diagnosed with diabetes at almost 500 000 people (6.5% of the population), where the highest prevalence is seen between the ages of 60 and 74 [8]. The most recent estimates point that a further 2.5% are with undiagnosed diabetes mellitus. In addition the official national statistical institute (NSI) reported aggregated data per classes of diseases for mortality of the population. Leading causes of mortality for the whole population for both genders are the diseases of circulatory organs, followed by neoplasms, and disease of the endocrine system metabolic disorders (NSI, www.nsi.bg). For males the leading cause remains the circulatory system diseases, followed by the respiratory diseases, and then neoplasms and endocrine disorders. However, few population-based studies have been conducted to assess the dynamics and relationship between diabetes and certain risk factors like age, gender, obesity and arterial hypertension. [9, 10]

To our knowledge, this is the first study aiming to evaluate life expectancy in patients with diabetes in Bulgaria and to compare it with life expectancy of the non-diabetic population in the country. The main study questions are:

1. What is the expected LE of the diabetic patients and that of the non-diabetic population for the period 2012–2015?
2. Is there a statistically significant difference in the expected LE in diabetic patients and that of the non-diabetic population for the period 2012–2015?
3. Is there a statistically significant difference in survival and mortality patterns among diabetic and non-diabetic population?
4. Is there a gender difference in life expectancy and survival for both cohorts?

## Methods

This is a retrospective observational population-based study of individuals diagnosed with diabetes, compared to the non-diabetic population in Bulgaria for the period from 2012 to 2015.

Demographic data about the population, expected LE, mortality, average age at time of death for the non-diabetic population and for diabetic patients (International classification of diseases (ICD) codes E00-E90), all-cause mortality, and gender differences were derived from the National Statistical Institute (NSI, www.nsi.bg) for the period 2012–2015. Data for mortality, and gender differences among people with diabetes for the same period were obtained from the National Diabetic Register (NDR, www.usbale.org). All people with diabetes type 1 and 2 were included, as well as separated by age groups and gender. The criteria for inclusion were diagnosis from ICD 10, belonging to codes E00-E90 confirmed in secondary care by endocrinologist.

The following statistics were done through MedCalc statistical software (MedCalc Software Ltd, Version 19.1.7)—Descriptive statistics of demographic data, expected Life-expectancy analysis, mortality analysis, as well as subgroup analysis, based on age and gender. Relative risks of death were calculated for each age group in 5 year age intervals, due to the nature of reporting by the NSI.

Average year of LE (YLE) was calculated as weighted value using the following formula:

AverageYLE=(Sumof(AgexNumberofdeaths))/Totalnumberofdeathsfortheyear

Kaplan-Meier survival analysis for different age and gender groups was carried out.

The survival probability at given moment of a time interval was calculated with the following formula:

Sp=(Numberofthealive–Numberofdeath)/Numberofaliveatbeginning

For each time interval, survival probability is calculated as the number of subjects surviving divided by the number of patients at risk.

Log Rank nonparametric test was applied to calculate the absolute hazard of death in the compared groups–with diabetes and in the non-diabetic population, using the formula:

$$\begin{array}{c} LogRank=\frac{{\left(Number\;of\;deaths\;in\;diabetic\;patients‐expected\;deaths\;in\;diabetic\;patients\right)}^{2}}{\left(expected\;number\;of\;deaths\;in\;diabetic\;patients\right)}\\ +\\ \frac{{\left(number\;of\;deaths\;in\;non‐diabetic\;population‐expected\;deaths\;in\;non‐diabetic\;population\right)}^{2}}{expected\;number\;of\;deaths\;in\;the\;non‐diabetic\;population} \end{array}$$

Statistical significance of the differences in life expectancy was tested with t-test and Chi square test, paired and independent t-tests. However, since statistics were done over the entire diabetic and non-diabetic populations, all results reached significance with small margin of error.

The risk of dying before entering the next age group was tested in both cohorts (with and without diabetes) for different age groups and genders by using the following formulas:

R1(k) = 1-S1(k) presents the risk of death for individuals with diabetes from age group k;

R2(k) = 1-S2(k) presents the risk of death for individuals without diabetes from age group k;

RR(k) = R1(k)/R2(k) presents the relative risk of death for age group k in both cohorts (with and without diabetes);

RR1(k) = R1(k-1)/R1(k) presents relative risk of death for individuals with diabetes in transition between age groups;

RR2(k) = R2(k-1)/R2(k) presents relative risk of death for individuals without diabetes in transition between age groups;

ARR(k) = R2(k)—R1(k) presents the absolute risk reduction in transition from age group k for individuals without diabetes to the same age group of individuals with diabetes;

Where: S1(k) and S2(k) is the probability of surviving of an individual in age group k with and without diabetes, respectively.

## Results

### Mortality and expected life expectancy

For the period 2012–2015 the total population of Bulgaria decreased by 130768 people, with the mortality remaining constant at approx. 1.5% throughout the observed period. A small increase of about 0.03% can be seen in 2015, compared to 2012. Male mortality comprised a higher percentage of death cases than female in all years, but no variations were observable. Female mortality showed higher variability with 2013 having the lowest percentage share of women (46.72%) and 2015 the highest (47.33%). The relative proportion of death cases in the diabetes group as a share of the total number of death cases in the non-diabetic population increased from 13.6% to 21% in 2015 (Table 1). Interestingly, female mortality percentages were higher for diabetics. Female diabetics had higher mortality than male in all observed years; however this discrepancy can be explained by the fact that females comprise a higher share of the diabetic population. To put these proportions into context, male mortality decreased from 2012 to 2015 (with 1.42%), whereas female mortality increased by 1.42%.

**Table 1:** Demographic data on population and mortality.

	2012	2013	2014	2015
**General and non-diabetic population**				
	***N (%)***	***N (%)***	***N (%)***	***N (%)***
Total number of population (NSI)^a^	**7284552**	**7245677**	**7202198**	**7153784**
Total recorded number of death cases in the general population (NSI) *(% of the population)*	**107558** *(1*.*48)*	**102899** (1.42)	**107410** *(1*.*49)*	**108299** *(1*.*51)*
Total number of male non-diabetic death cases (NSI) *(% of total deaths)*	**56702** *(52*.*72)*	**54827 (***53*.*28)*	**56540** *(52*.*64)*	**57040** *(52*.*68)*
Total number of female non- diabetic death cases (NSI) *(% of total deaths)*	**50856** *(47*.*28)*	**48072** *(46*.*72)*	**50870** *(47*.*63)*	**51259** *(47*.*33)*
**Diabetic population**				
Total number of patients with diabetes (NDR)^b^ *(% of total population)*	**425262** *(5*.*83)*	**441217** *(6*.*08)*	**456165** *(6*.*33)*	**467458** *(6*.*53)*
Patients with type 1 diabetes (NDR) *(% of total diabetic population)*	**28108** *(6*.*61)*	**27886** *(6*.*32)*	**27193** *(5*.*96)*	**26259** *(5*.*62)*
Patients with type 2 diabetes (NDR) *(% of total diabetic population)*	**397154** *(93*.*39)*	**413331** *(93*.*68)*	**428972** *(94*.*04)*	**441199** *(94*.*38)*
Total number of patients with diabetes (NDR)–male *(% of total diabetic population)*	**190794** *(44*.*87%)*	**198434** *(44*.*97%)*	**200426** *(43*.*94)*	**206097** *(44*.*09%)*
Total number of patients with diabetes (NDR)–female *(% of total diabetic population)*	**234468** *(55*.*13)*	**242783** *(55*.*03)*	**255739** *(56*.*06)*	**261361** *(55*.*91)*
Total number of deaths in the diabetes groups *(% of all death cases)*	**14733** *(13*.*69)*	**18823** *(18*.*29)*	**21272** *(19*.*8)*	**23097** *(21*.*26)*
Number of deaths in type 1 diabetes group (NDR) *(% of all death cases)*	**1318** *(1*.*22)*	**1474** *(1*.*43)*	**1492 *(****1*.*39)*	**1456** *(1*.*34)*
Number of deaths in type 2 diabetes group (NDR) *(% of all death cases)*	**13415** *(12*.*47)*	**17349** *(16*.*86)*	**19780** *(18*.*41)*	**21641** *(19*.*98)*
Total number of deaths in the diabetes group—male *(% of all diabetes death cases)*	**7202** *(48*.*88)*	**9120** *(48*.*45)*	**10151** (*47*.*72*)	**10961** *(47*.*46*
Total number of deaths in the diabetes group—female *(% of all diabetes death cases)*	**7531** *(51*.*12)*	**9703** *(52*.*55)*	**11121** *(52*.*28)*	**12136** *(52*.*54)*

a) NSI—National statistical institute

b) NDR–National diabetes registry

The expected average LE in type 2 diabetes patients, however, is higher in comparison with the LE in the non-diabetic population, while the average LE for type 1 patients is almost 4 years lower. This could be explained by the mean age of registering the type 2 diabetes patients in the registry which corresponds with the mean age of diagnosis of the diabetes type 2 population– 65 years of age. After confirming the diagnosis by the endocrinologist, the patients become more frequently monitored compared to the non-diabetic population which allows diagnosis and treatment of diabetes-related complications, as well as shorter time to develop complications compared to type 1 diabetics. (Table 2).

**Table 2:** Expected average life expectancy.

	2012	2013	2014	2015
LE non-diabetic population	74.7	74.5	74.7	74.8
LE in the diabetes population	73.96	73.96	74.18	74.64
LE in type 1 diabetes	70.02	69.77	70.43	70.96
LE in type 2 diabetes	74.20	74.34	74.59	75.19
LE non-diabetes population-male	71.2	71.1	71.2	71.3
LE in type 1 diabetes–male	67.45	67.63	67.42	67.97
LE in type 2 diabetes–male	72.48	72.30	72.59	73.22
LE in the male diabetes population	70.91	70.89	71.16	71.50
LE non-diabetic population-female	78.3	78.0	78.2	78.4
LE in the female diabetes population	75.55	75.95	76.22	76.80
LE in type 1 diabetes–female	72.96	71.92	73.55	74.06
LE in type 2 diabetes–female	75.77	76.26	76.41	76.95

Average life expectancy in 2015 in the non-diabetic population is around 74.8 years with longer life expectancy for the female than male population (78 years vs 71) (Table 2). Patients with Type 1 DM and with Type 2 DM are expected to have an average life of 70.96 and 75.19 years at the end of observed period. The combined diabetic life expectancy is 74.64 years—comparable to the life expectancy in the general population. Stratifying the diabetic and non-diabetic population by type of diabetes and sex provides some interesting insight into the dynamics. For the type-2 male population LE is higher than that of the non-diabetic male population, however, in the female non-diabetic population it is higher than the life expectancy for type-2 diabetic females. Despite this, type-2 diabetic women saw a steady increase in life expectancy from 75.77 years to 76.95 years, with the gap closing from 2.6 years in 2012 to 1.45 years. This is also observable in the male type-2 diabetic population–both sexes saw an increase in life expectancy, whereas the LE in the non-diabetic population remained constant. Type-1 diabetic patients have the lowest LE with the male, female and combined numbers being 67.97, 74.06, and 70.96 years in 2015, respectively. All observed differences reached statistical significance, due to the analysis being based on entire populations.

The mortality for both the diabetic and non-diabetic populations per age group is presented in Table 3. Mortality presented as a percent, decreased or remained constant for all age groups, apart from the age 80+. Most notably for the ages 70–74 mortality decreased significantly from 16.4% (2012) to 15.7% (2015) for diabetic population, as well as for the ages 75–79 it decreased from 20.9% (2012) to 17.9%). This decrease in younger ages is compensated with increased mortality in the age group 85+ (from 12.9% in 2012 to 23.9% in 2015).

**Table 3:** Number of death cases according to age groups in diabetic and non-diabetic population.

Age group	2012 Diabetics n (%)	2012 Non diabetic n (%)	2013 Diabetics n (%)	2013 Non Diabetics n (%)	2014 Diabetics n (%)	2014 Non diabetic n (%)	2015 Diabetics n (%)	2015 Non diabetics n (%)
0	0							
1–4	0	118 (*0*.*11)*	0	115 *(0*.*11)*	0	88 *(0*.*8)*	0	79 *(0*.*07)*
5–9	*0*	*68 (0*.*06)*	0	66 *0*.*06)*	0	55 *(0*.*05)*	0	69 *(0*.*06)*
10–14	0	76 *(0*.*07)*	0	66 *(0*.*06)*	0	62 *(0*.*06)*	0	59 *(0*.*05)*
15–19	2 *(0*.*01)*	170 *(0*.*16)*	*0*	*138 (0*.*13)*	0	150 *(0*.*14)*	4 *(0*.*02)*	150 *(0*.*14)*
20–24	3 *0*.*02)*	282 *(0*.*26)*	4 *(0*.*02)*	302 *(0*.*29)*	7 *(0*.*03)*	263 *0*.*24)*	4 *(0*.*02%)*	240 *(0*.*22)*
25–29	6 *(0*.*04)*	332 *0*.*31)*	9 *(0*.*05)*	334 *(0*.*32)*	3 *(0*.*01)*	321 *(0*.*3)*	13 *(0*.*06)*	350 *(0*.*32)*
30–34	12 *(0*.*08)*	492 *(0*.*48)*	24 *(0*.*13)*	508 *(0*.*49)*	20 *(0*.*09)*	517 *(0*.*48)*	18 *(0*.*08)*	502 *(0*.*46)*
35–39	40 *(0*.*27)*	822 *(0*.*76)*	40 *(0*.*21)*	769 *(0*.*75)*	43 *(0*.*02)*	*890 (0*.*83)*	46 *(0*.*19)*	766 *(0*.*71)*
40–44	75 *(0*.*51)*	1288 *(1*.*2)*	106 *(0*.*56)*	1323 *(1*.*29)*	103 *(0*.*48)*	1349 *(1*.*26)*	79 *(0*.*34)*	1308 *(1*.*21)*
45–49	144 *(0*.*98)*	2005 *(1*.*86)*	177 *(0*.*94)*	2058 *(2*.*0)*	199 *(0*.*94)*	2091 *(1*.*95)*	223 *(0*.*97)*	2166 *(2*.*0)*
50–54	325 *(2*.*21)*	3686 *(3*.*43)*	435 *(2*.*31)*	3463 *(3*.*37)*	493 *(2*.*32)*	3748 *(3*.*49)*	430 *(1*.*86)*	3456 *(3*.*19)*
55–59	665 *(4*.*5)*	5669 *(5*.*3*)	902 *(4*.*8)*	5519 *(5*.*36)*	1010 *(4*.*75)*	5630 *(5*.*24)*	927 *(4*.*01)*	5506 *(5*.*08)*
60–64	1339 *(9*.*09)*	8479 *(7*.*9)*	1588 *(8*.*4)*	7962 *(7*.*74)*	1795 *(8*.*4)*	8389 *(7*.*8)*	1861 *(8*.*06)*	8100 *(7*.*48)*
65–69	1920 *(13*.*03)*	10044 *(9*.*34)*	2561 *(13*.*6)*	10215 *(9*.*93)*	2845 *(13*.*4)*	10822 *(10*.*1)*	2971 *(12*.*9)*	11145 *(10*.*3)*
70–74	2412 *(16*.*4)*	12116 *(11*.*3)*	2903 *(15*.*4)*	11338 *(11*.*02)*	3228 *(15*.*2)*	11760 *(10*.*9)*	3635 *(15*.*7)*	12236 *(11*.*3)*
75–79	3079 *(20*.*9)*	17806 *(16*.*6)*	3751 *(19*.*9)*	16381 *(15*.*9)*	3909 *(18*.*4)*	16051 *(14*.*9)*	4154 *(17*.*9)*	15230 *(14*.*06)*
80–84	2801 *(19*.*02)*	20066 *(18*.*7%)*	3660 *(19*.*4)*	19081 *(18*.*6)*	4263 *(20*.*04)*	20204 *(18*.*8)*	4625 *(17*.*8)*	20668 *(19*.*08)*
85+	1910 *(12*.*9)*	23503 *(21*.*9)*	2663 *(14*.*2)*	22733 *(22*.*1)*	3354 *(15*.*8)*	24443 *(22*.*8)*	4107 *(17*.*8)*	25835 *(23*.*9)*
Total	14733	107558	18823	102899	21272	107410	23097	108299

**Fig 1:**
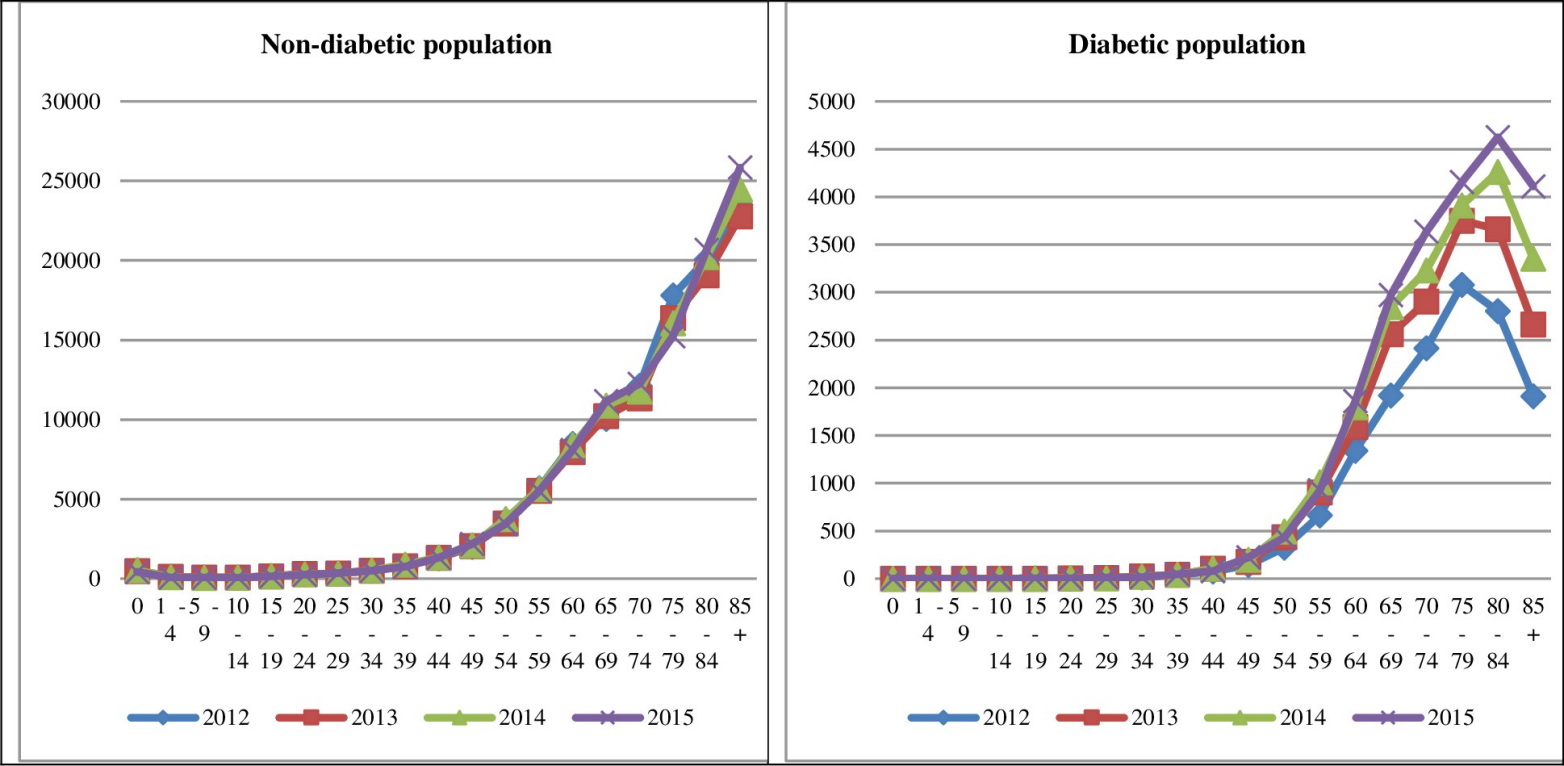
Total mortality for all years in the observed period in the non-diabetic and diabetic population.

For the diabetes population, male mortality dominates up to the age of 75, whereas for the non-diabetic population, male mortality is higher up to the age of 80+, beyond which respective ages female mortality overtakes male, indicating longer life span for women (Table 4). Fig 1 presents the tendencies in mortality for the entire observed period for both the non-diabetic and diabetic population, as well as individually for men and women. As expected, for the non-diabetic population, as age progresses so do the number of deaths recorded each year, showing a clear growing trend. The trend for the diabetic population shows an initial growth up to a certain age, after which the number of recorded deaths drops and a reduction in mortality is observed. What is interesting is that for 2012 that highest mortality numbers occurred in the age group 75–79, whereas for 2015 the highest number of deaths occurred for the ages 80–84. In 2012 the highest recorded number of deaths was 3079 between the ages of 75 and 79, and for 2015, 4623 diabetic patients died between the ages of 80 to 84 years, with a subsequent drop in mortality for the next age group (Table 3).

**Table 4:** Distribution of deaths, according to age and gender in diabetic and non-diabetic populations.

Age group	2012 People with diabetes		2012 Non-diabetic population		2013 People with diabetes		2013 Non-diabetic population		2014 People with diabetes		2014 Non-diabetic population		2015 People with diabetes		2015 Non-diabetic population	
	male	female	male	female	male	female	male	female	male	female	male	female	male	female	male	female
0	0	0	327	209	0	0	283	206	0	0	284	233	0	0	245	189
1–4	0	0	65	53	0	0	70	45	0	0	46	42	0	0	39	40
5–9	0	0	36	32	0	0	39	26	0	0	30	25	0	0	47	22
10–14	0	0	52	24	0	0	41	25	0	0	41	21	0	0	35	24
15–19	1	1	123	46	0	0	96	42	0	0	18	132	2	2	104	44
20–24	3	0	218	64	1	3	231	68	5	2	186	75	1	2	170	67
25–29	5	1	250	81	4	5	238	91	3	0	222	99	10	2	257	90
30–34	8	4	352	136	15	9	368	131	14	6	366	145	12	6	364	132
35–39	26	14	541	267	19	21	528	220	29	14	648	228	32	18	523	229
40–44	54	21	901	366	80	26	893	404	70	33	875	441	59	17	877	411
45–49	105	39	1393	573	124	53	1475	530	150	49	1500	542	148	74	1499	592
50–54	218	107	2654	925	296	139	2466	858	328	165	2594	989	276	154	2484	818
55–59	455	210	4093	1366	597	305	3949	1265	644	366	4008	1256	594	331	3849	1324
60–64	808	531	5882	2066	966	622	5669	1671	1099	696	5794	1899	1120	729	5666	1693
65–69	1046	874	6536	2634	1445	1116	6779	2320	1611	1234	7222	2426	1705	1250	7379	2500
70–74	1237	1175	7030	3911	1483	1420	6742	3176	1574	1654	6850	3256	1864	1766	7268	3197
75–79	1333	1746	8906	7154	1701	2050	8363	5968	1755	2154	8134	5763	1871	2280	7773	5174
80–84	1155	1646	8779	9641	1440	2220	8266	8595	1647	2616	8791	8797	1801	2822	8986	8858
85 +	748	1162	8564	13777	949	1714	8331	12728	1222	2132	8931	13380	1466	2683	9475	13719
**Total**	7202	7531	56702	43325	9120	9703	54827	38369	10151	11121	56540	39749	10961	12136	57040	39123

**Fig 2:**
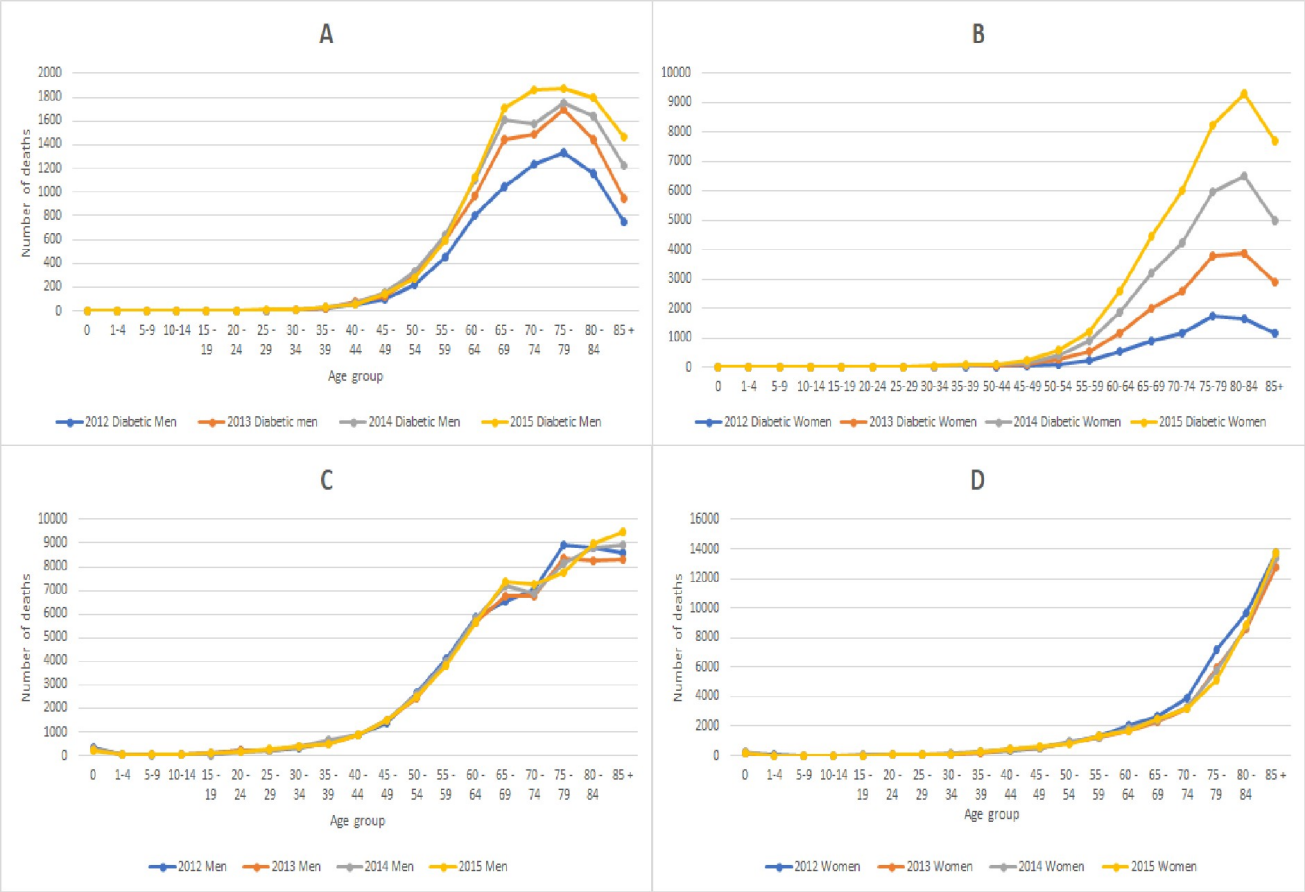
Mortality trends for each observed year in **A)** All Diabetic Male, **B)** All Diabetic Female, **C)** All Non-Diabetic Male, **D)** All Non-Diabetic Female.

This drop in mortality for the diabetic population can be observed in both males and females for each year, showing a clear trend (Fig 2).

The data from the National diabetes register indicates that for diabetic women, life expectancy is increasing each consecutive year. The highest number of deaths recorded in 2012 for both diabetic men and women were between the ages of 75 and 79, with 1333, and 1746 people dying in that age group, respectively. By 2015, however, the highest point of female mortality was in the age group 80–84 with 2822 recorded cases. Men saw no change in status, with 1864 dying between the ages of 70–74 and 1871 between the ages of 75–79. What this data indicates is that women have a higher probability of reaching ages 80+ than men and suggest that life expectancy for diabetic women is increasing.

### Survival analysis

**Fig 3:**
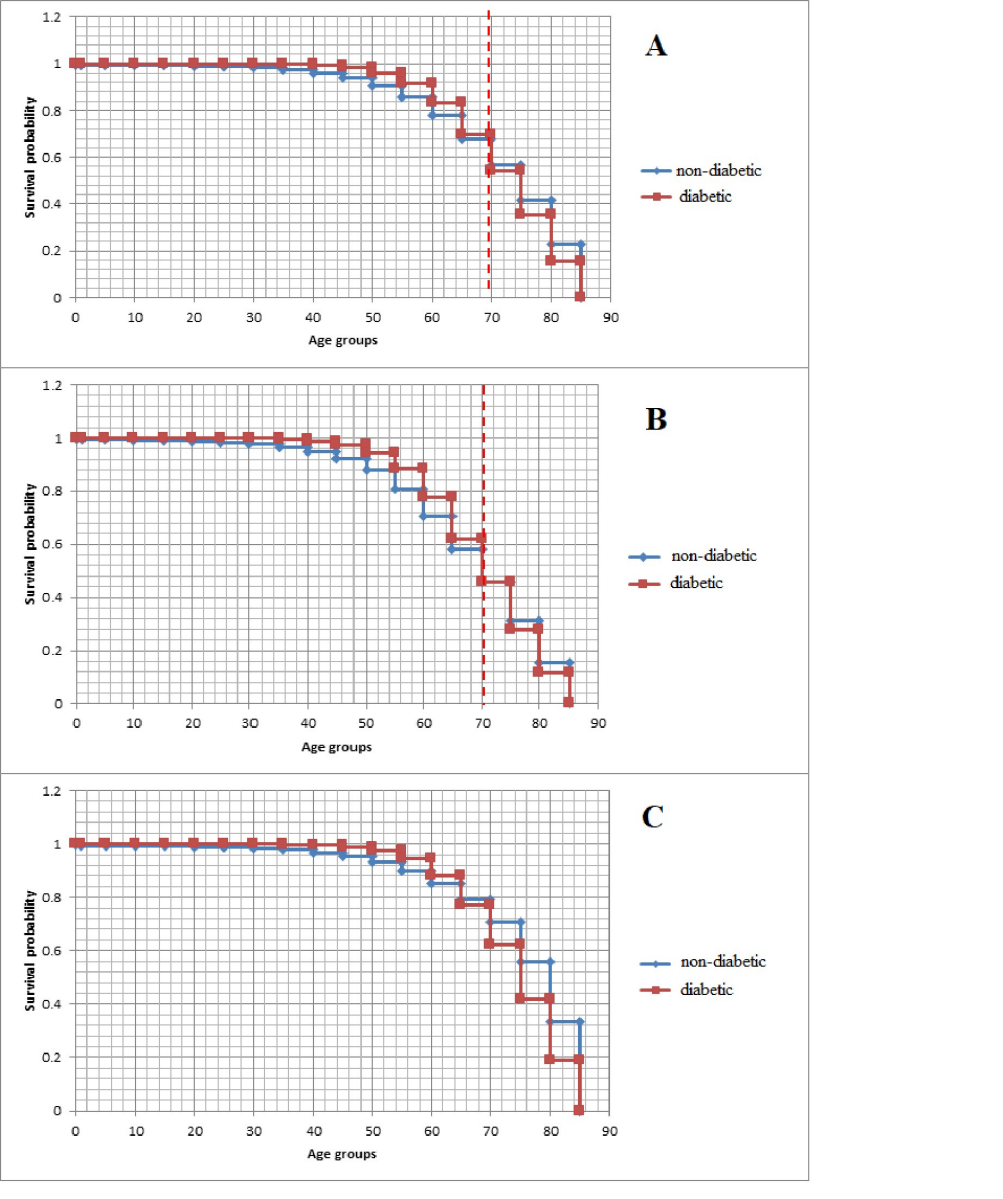
Kaplan-Meier survival curves for **A)** The entire diabetic and non-diabetic population, **B)** The entire Male diabetic and non-diabetic population, and **C)** the entire female diabetic and non-diabetic population.

Throughout most of their lives, diabetic patients have a higher probability of surviving to the next age group than the non-diabetic population. This is mainly due to the high infant, child and adolescent mortality observed in the non-diabetic population. Nonetheless, there is a clear tendency of decreasing survival rates for diabetic patients: Up to the age of 70 male diabetics consistently has higher probability of surviving to the next age group (Table 5). For diabetic women this is true up to the age of 65. The observed discrepancy in survival probabilities can be attributed to the high male mortality in the non-diabetic population. As shown in Table 2, women are expected on average to live 7.1 more years. The Kaplan-Meier survival curves clearly illustrate these differences (Fig 3). When comparing the non-diabetic to the entire diabetic population, despite being closely matched, diabetics have a slightly higher survival probability than non-diabetics up to the age of 70. Diabetic survival probability decreases between the ages of 65–69, and is equalized at age 70, before going lower. The same is observed when analyzing only the male populations, however, for women survival probability begins decreasing between the ages of 60–64, equalizing at 65 years of age, and subsequently falling well below the survival probability in the non-diabetic female population.

**Table 5:** Difference between the probabilities for survival.

**Age groups**	**Difference between the probabilities for survival in diabetic and nondiabetic population (S1-S2)-all**	**Dominant zones for survival in diabetic and nondiabetic population (S1 and S2) –all**	**Difference between the probabilities for survival in diabetic and nondiabetic population (S1-S2) male**	**Dominant zones for survival in diabetic and nondiabetic population (S1 and S2)-male**	**Difference between the probabilities for survival in diabetic and nondiabetic population (S1-S2)—female**	**Dominant zones for survival in diabetic and nondiabetic population (S1 and S2)—female**
0	0,005674		0,006069		0,00521	
1–4	0,006823		0,007241		0,00633	
5–9	0,007561		0,008051		0,00699	
10–14	0,008316		0,008952		0,00757	
15–19	0,009968		0,010672		0,00914	
20–24	0,012806	S1>S2;	0,014641	S1>S2;	0,01068	S1>S2;
25–29	0,016159	HR 87;	0,019089	HR^c^82;	0,01273	HR^c^ 77;
30–34	0,020795	p = 0,000	0,025245	p = 0,000	0,0155	p = 0,000
35–39	0,027464		0,033784		0,01972	
40–44	0,036891		0,044251		0,02743	
45–49	0,049114		0,058627		0,03605	
50–54	0,0639		0,077142		0,04446	
55–59	0,072976		0,088482		0,04698	
60–64	0,064155		0,083149		0,02895	
65–69	0,023874		0,045827		-0,02001	
70–74	-0,03112	S1<S2;	-0,00288	S1<S2;	-0,08423	S1<S2
75–79	-0,07701	HR 127;	-0,03951	HR 120;	-0,13765	HR 137;
80–84	-0,08827	p<0,0001	-0,04759	p<0,0001	-0,1439	p<0,0001
85 +	0		0		0	

S1 –probability for survival in diabetic patients

S2—probability for survival in the general population

HR–hazard ratio (%)

The log-rank test (Table 6) showed an overall 12% higher hazard of death in diabetics than the non-diabetic population. Interestingly male diabetics only have a 2% higher hazard, while female diabetics have a 26% higher risk. All observed differences were statistically significant with p values < 0.001.

**Table 6:** Results from logrank test comparing survival data and death risks in diabetic and non-diabetic population.

Log-rank test result			
	all	male	Female
Log–Rank	539,47	11,26	1755,30
Degree of Freedom	1	1	1
P-value	0,000	0,001	0,000
Hazard Ratio (%)	112	102	126

### Relative risk of death

Age adjusted risks of death show meaningful changes after the age of 60 for the diabetic population, coinciding with the age of diagnosis. For the age group 60–64 the risk of death in the diabetic population is 0.09228 vs. 0.08981 for the non diabetic population. The relative risks are 197% for diabetics and 147% for non-diabetics for the same age group and an absolute risk reduction of -0.25%. As potential comorbidities and diabetes complications develop, the risk for the diabetic population increases, with the highest being 0.56 for the age group 80–84 vs 0.43 for the non-diabetic population in the same age group. This translates to an absolute risk reduction of -12.7%. At ages 80–84 the total relative risk (RR) of death is 129%, 159% for diabetics and 171% for non-diabetic. Subgroup analysis by sex shows women consistently have a higher risk of death (with one outlier) with a maximum of 2.68 (268%) in the age group 30–34. This risk is similar to that of ages 50–54, where the risk in diabetic women is 264%. The highest calculated relative risk was for diabetic men ages 20–24 where analysis showed a 3 times higher risk of death (333%) (Table 7).

**Table 7:** Risks for death in the diabetes and non-diabetic group.

	Risks for death in the diabetic and non-diabetic group						Risks for death in the male diabetic and non-diabetic						Risks for death in female diabetes and non-diabetic					
Age group	R1	R2	RR in %	RR1 in %	RR2 in %	ARR in %	R1	R2	RR in %	RR1 in %	RR2 in %	ARR in %	R1	R2	RR in %	RR1 in %	RR2 in %	ARR in %
	0	0				0,00	0	0				0,00	0	0				0,00
0	0	0,00568	0	-	-	0,57	0	0,00616	0	-	-	0,62	0	0,00521	0	-	-	0,52
1–4	0	0,00116	0	-	20	0,12	0	0,00118	0	-	19	0,12	0	0,00113	0	-	22	0,11
5–9	0	0,00074	0	-	64	0,07	0	0,00082	0	-	70	0,08	0	0,00066	0	-	58	0,07
10–14	0	0,00079	0	-	106	0,08	0	0,00097	0	-	118	0,10	0	0,00059	0	-	90	0,06
15–19	0,00008	0,00174	4	-	221	0,17	0,00008	0,00233	3	-	240	0,23	7,41E-05	0,00166	4	-	281	0,16
20–24	0,00023	0,00310	7	300	178	0,29	0,00027	0,00433	6	333	186	0,41	0,00017	0,00172	10	233	104	0,15
25–29	0,00039	0,00380	10	172	123	0,34	0,00059	0,00513	11	220	118	0,45	0,00020	0,00227	9	114	132	0,21
30–34	0,00095	0,00568	17	239	149	0,47	0,00131	0,00768	17	223	150	0,64	0,00062	0,00343	18	313	151	0,28
35–39	0,00217	0,00904	24	229	159	0,69	0,00284	0,01175	24	217	153	0,89	0,00166	0,00598	28	268	174	0,43
40–44	0,00468	0,01454	32	215	161	0,99	0,00706	0,01828	39	249	155	1,12	0,00240	0,01033	23	145	173	0,79
45–49	0,00962	0,02279	42	206	157	1,32	0,01425	0,03036	47	202	166	1,61	0,00534	0,01440	37	222	139	0,91
50–54	0,02199	0,03900	56	229	171	1,70	0,03067	0,05307	58	215	175	2,24	0,01411	0,02345	60	264	163	0,93
55–59	0,04682	0,06028	78	213	155	1,35	0,06481	0,08383	77	211	158	1,90	0,03068	0,03485	88	218	149	0,42
60–64	0,09228	0,08981	103	197	149	-0,25	0,12083	0,12752	95	186	152	0,67	0,06733	0,05079	133	219	146	-1,65
65–69	0,15903	0,11980	133	172	133	-3,9	0,19988	0,16949	118	165	133	-3,04	0,12527	0,07213	174	186	142	-5,31
70–74	0,22364	0,15007	149	141	125	-7,4	0,26491	0,20048	132	133	118	-6,44	0,19254	0,10654	181	154	148	-8,60
75–79	0,35228	0,25317	139	158	169	-9,9	0,38975	0,30596	127	147	153	-8,38	0,32626	0,21187	154	169	199	-11,44
80–84	0,56053	0,43347	129	159	171	-12,7	0,57950	0,48040	121	149	157	-9,91	0,54746	0,40104	137	168	189	-14,64
85 +	1	1	100	178	231	0,00	1	1	100	173	208	0.00	1	1	100	183	249	0,00

## Discussion

The current study, using data from the National Diabetes Registry in Bulgaria, found that diabetic patients have equal, and in some cases- higher, average life expectancy in comparison to the general population. However, our findings demonstrated that type 1 diabetic patients had an estimated life expectancy (LE) loss of approximately 3 years. These results could probably be explained by the early type 1 diabetes diagnosis, earlier onset, and earlier development of micro and macro vascular complications in this group of patients in comparison with type 2 diabetes. The results reflect the actual life expectancy and the death rate among the diagnosed with diabetes and non-diabetic Bulgarian population.

The dynamics of the disease, survival and life-expectancy, however seem to be more complicated. Our analysis found that diabetic men can be expected to live 0.2 years longer than men in the non-diabetic population, while diabetic women have a 1.6 years lower life expectancy than the non-diabetic female population in 2015. Moreover, our analysis showed that life expectancy in the male non-diabetic population is significantly lower than that of the female population with a difference of 7.1 years in favor of women, thus making non-diabetic men have the same life expectancy as diabetic men. Historically men have had a shorter life span than women, as confirmed by the 2019 World Health Organization’s life expectancy report which can be attributed to the fact that men are more likely to be engaged in unhealthy behaviors such as smoking [11, 12], and alcohol consumption [13], as well as have probably riskier occupations with the Bureau of Labor statistics in the USA documenting a 12 times higher risk of fatal workplace injuries in men [14]. However, the differences in our findings could also be attributed by the disparity in the ratio of male to female diabetic patients. Contrary to reports by the International Diabetes Federation [IDF] and World Diabetes Foundation, where the prevalence of diabetes is higher in men, our study revealed a larger percentage share of women being afflicted by the disease [15].

Survival probabilities for diabetics are higher among those in the age group up to 65 years and equalizes to that of the non-diabetic population around the age of 70. In the group over 70 years of age the LE is higher in the non-diabetic population. The observed decline in survival probability could be explained by the age of diagnosis of type-2 diabetes. However, our analysis did show a gradual increase in life expectancy and reduction in mortality, particularly for the female diabetic population for the period 2012–2015. Similarly, Muschik et al.(2017) concluded that the life expectancy among diabetics in Lower Saxony, Germany had been increasing for the period 2005–2014, but the paper has not made any comparative analysis of diabetic patients with general population [7]. Logically, diabetes-related mortality rates rise as the age of patients progresses, which have been demonstrated in previous studies [4]. The mean age of diagnosis and life expectancy for the general population should be noted. In our study type-2 patients were diagnosed around the age of 65 making additional years of life expected equal to 9.64. In 1985 Krolewski et al. reported that nephropathy took approximately 10 years to develop in patients with type-2 DM, while the 10-year post trial follow up of UKPDS reported a relative risk reduction in myocardial infarction rates, indicating that intensive therapy may play a benefit in improved cardiovascular outcomes [16, 17]. Our results support the hypothesis that good glucose control can positively affect life expectancy and our data is rooted in real-world practice. In our study mortality rates among diabetic patients over 70 years of age showed a decrease, with a corresponding increase in LE, most notably in type-2 diabetics, where in 2015 the average life expectancy had increased by almost a year to 75.1s years. Comorbidities and complications do play a role, however, and the log rank test revealed an overall 12% greater hazard of dying in diabetics, which is statistically significant (p<0.0001). Opposite results are observed among patients up to the ages of 70, where the constructed life tables and differences in survival probabilities showed a hazard ratio of 0.87 (87%), meaning that diabetics do not have a higher risk than the non-diabetic population (p<0.0001). A Canadian study showed that life expectancy among diabetic patients decreases with advancement of age, similar to our results. Moreover, the loss in LE at birth for women diagnosed with diabetes in the Canadian study was 10.1 years, with the loss of LE at age 80 being 2.6 years. We did, however, confirm that females in the non-diabetic population tend to live longer lives, although we did not analyze health-adjusted life-expectancy (HALE) [18]. Our study uses real-world data from two comprehensive national databases, stratifying them by age, type of diabetes and sex, which have been identified as potential confounders and revealed a striking similarity in life-expectancies, well below the 10-year gap documented, with a tendency of decreasing mortality and increasing life.

We established significant differences [19] when comparing our results to findings of a similar study aiming to reveal current life expectancy in people with and without type 1 diabetes in Scotland. In that study conducted among a national population with type 1 diabetes, a significant loss of life was established– 11,1 years which is nearly 4 times higher in comparison to our observed values– 3.68 years loss of life for type-1 diabetics. The study also documented a wide gap in survival, where 76% of men and 83% of women in the non-diabetic population survived to age 70 in comparison to 47% and 55% with type 1 diabetes, respectively. In our analyzed populations, for every year in the observed period, the majority of deaths occurred after the age of 70, whereas between 22.6 and 28.5% of the deaths occurred before the age of 60. The differences could be explained by the different time periods of both studies: the Scottish study is based on local data collected between 2008 and 2010, whereas our study included the time horizon during 2012–2015. Taking into account the progress of treatment approaches and improved medical care, or glucose delivery devices, we would expect the life expectancy in this group of patients to increase [20]. This is supported, in part, by another study we conducted, evaluating disease control, where the percentage of patients achieving glycosylated hemoglobin levels of under 7 increased from 24.6% in 2012 to 32.44% in 2016 [22]. In Australia, patients with diabetes also achieved a high life expectancy of 80.2 years, which however, was still 3.2 years lower than that of the general population [21]. Our findings need to be put in context for Bulgaria, since the LE in the general population is well below the expected one in developed countries– 74.8 (as of 2015) years, and the average age of diabetes patients is 65. Typically, diagnosis occurs at this age and within the next couple of years patients start to develop complications thereby increasing the risk of death. This is reflected in the data, where the survival probability between the non-diabetic and diabetic population is equalized at the age of 70–74 and subsequently rises higher for the diabetics. The fact that diabetes prevalence was higher in women might also play a role, however, we cannot comment on why this prevalence is different from other reported ones. A normal life expectancy in a cohort of type 2 diabetes patients was determined in the Netherlands when a comparison with the general population was performed for the period 2001–2007 which is very similar to our results. In contrast to our study, it also analyzed comorbidities as confounders such as history of cardiovascular disease and albuminuria. Ensuring an adequate control over environmental factors and a complex medical care for diabetes patients could bring a normal life to them, and diminish the risk of decreased LE. [20]. Due to the specificity of Bulgarian death certificates, where comorbidities are not always listed, we could not perform any comorbidity adjusted analyses. The latter also concerns and the information published by the National Statistical Institute.

What further contributes to the hypothesis that care on a national level has improved is the Body Mass Index of diabetic patients. Two epidemiologic studies, whose results were published 2015 found that in 2006 44.2% of patients had a BMI ≥ 30, while in 2012 that percentage was 51% [9], whereas analysis of the diabetes register, published in 2019 reveals that in 2017 this percentage was 36.44% [22]

Limitations of our study include lack of analysis of the influence of various factors such as cardiovascular diseases, tobacco consumption, levels of glycosylated hemoglobin (HbA_1c_), duration of diabetes, body mass index and most importantly, the presence of comorbidities on the LE and mortality rates among diabetic patients in Bulgaria. Although such comparisons and detailed factor analyses, which are performed in a number of other studies,[23, 24, 25, 26]could provide important information about the individual prognosis and health gains that could be achieved if some risk factors are eliminated or reduced, as well as eliminate the selection bias present in our cohorts, it was far beyond our ability to conduct them, based on currently available data. We have made an attempt to segregate the diabetic and nondiabetic populations as well as stratify them by sex and age, and recognize that there might be the problem of selection bias emanating from the lack of co-morbidity information. But as was clarified—our primary goal was to analyze the mortality data as they appear in the official statistical reports for both cohorts. Underlying factor analysis goes far beyond this primary goal. Despite these shortcomings, data from the diabetes register clearly show an increase in the life expectancy of diabetics, which outpaces the growth in life expectancy in the non-diabetic population, reported by the national statistical institute. We also recognize that the leading role of cardiovascular, pulmonary and oncology diseases might be substantially high for a patient with a comorbidity history of having diabetes but by separating both cohorts of diabetics and non-diabetics we are trying to give more light on the mortality due to diabetes as a primary factor. As was pointed out in the introduction, the leading three causes of death according to NSI are “diseases of the circulatory organs and system”, followed by “neoplasms” and “endocrine system and metabolic disorders”. NSI data also show that “endocrine system” mortality lags behind mortality due to “neoplasms” up until the age of 75, after which it becomes the 2^nd^ leading cause of death for the country. Unfortunately the NSI shows aggregate data, which does not provide an opportunity for more detailed stratification and analysis which we tried to do in our study.

A further limitation is the lack of data on patients with undiagnosed diabetes. By some estimates, there are approximately 2.5 percent of diabetic cases in Bulgaria which are undiagnosed–a fact that might further complicate the segregation. Perhaps a higher percentage of men remain with undiagnosed Diabetes Mellitus, hence the low prevalence in this population, which would put them in the non-diabetic population for the analysis, introducing a potential source of bias. If international data is also relevant in Bulgaria, a further 10% of the male population could have diabetes.

There are, however, certain studies that address the role of certain risk factors in diabetes control. The study by Borissova et al. reported that hypertension was present in 80.5% of the studied population [9, 10]. We can, therefore, suspect that a major driver in mortality would be cardiovascular (CV) events, associated with both diabetes and hypertension. This is indicated in the percentage of costs that the NHIF has paid for CV events– 26% for Heart Failure, 12% for Myocardial Infarction, and 10% for stroke. [27]. Cardiovascular events are also prevalent in the general population. A study in 2008 reported that for the ages between 45–64, 48.7% were at a high risk and a further 10.1% were at an excessive high risk of developing a CV event, measured via the European Systematic Coronary Risk Evaluation (SCORE, HeartScore®). Furthermore, the study reported that in men aged 64 and over, the prevalence of excessive risk was 46.6% [28], clearly indicating that CV mortality plays a role in the short Life Expectancy for the general population. In 2017 the National Statistical Institute reported that 65% of all deaths are due to a cardiovascular event, unfortunately the death certificates only mention the cause of death, without indicating if any underlying disease is present.

Therefore, the analytical exercise carried on in this paper is highly contaminated by the presence of selection bias, but, as was mentioned, further detailed studies are needed to deeper analyze the underlying factors and this study addresses an acute problem, which we hope would start a discussion around these issues. It still stands to reason that both for type 1 and type 2 diabetes, female life expectancy is increasing, and the data clearly show that this is in contrast to the stagnant LE in the female non-diabetic population. The question then should focus on what are the reasons for this, which is a topic for further analysis, and further data collection.

The current study raises questions regarding the way mortality is documented in Bulgaria. There is a lack of data in death records about death due to chronic or acute diabetes complications (or lack of data for comorbidity). A need for more strict documentation of the patients with diabetes is highlighted. Only then precise further analyses for assessing the influence of different variables on the LE could be performed.

## Conclusion

Patients with type 2 DM have a longer life-expectancy than patients with type-1 DM and overall Diabetes life expectancy equals that of the non-diabetic population, which could suggest improved disease control and its associated complications in Bulgaria. Male diabetics show slightly longer life expectancy than their counterparts in the non-diabetic population, but a marginal gain of 0.6 years for the entire observed period. Life expectancy in diabetic women increased by 1.3 years, which was not observed in the non-diabetic population. Prevalence of diabetes was higher for women. Improved diabetes control may explain this gain in life; however other studies are needed to confirm this.

## Supporting information

S1 Data (XLS)

## References

[1] World Health Organization: Global report on diabetes. 2016; 1–88. Available from: http://apps.who.int/iris/bitstream/handle/10665/204871/9789241565257_eng.pdf;jsessionid=DE86065D8957A520439F6D8228C79DA1?sequence=1.

[2] Anwer Z, Sharma PK, Garg VK, Kumar N, Kumari A. Hypertension management in diabetic patients. Eur Rev Med Pharmacol Sci. 2011; 15 (11): 1256–1263. 22195357

[3] Leal J, Gray AM, Clarke PM. Development of life-expectancy tables for people with diabetes type 2. Eur Heart J. 2009; 30 (7): 834–839. 10.1093/eurheartj/ehn567 19109355 PMC2663724

[4] Iocara S, Sava E, Georgescu O, Sirbu A, Fica S. Recent diabetes-related mortality trends in Romania. Acta Diabetologica. 2018; 55 (8): 821–826. 10.1007/s00592-018-1156-5 29774467

[5] Sikdar KC, Wang PP, MacDonald D, Gadag VG. Diabetes and its impact on health-related quality of life: a life table analysis. Qual Life Res. 2010; 19: 781–787. 10.1007/s11136-010-9641-5 20349211

[6] Bardenheier BH, Lin J, Zhuo X, Ali MK, Thompson TJ, Cheng YJ, et al Disability-Free Life-Years Lost Among Adults Aged ≥50 Years With and Without Diabetes. Diabetes Care. 2016; 39 (7): 1222–1229. 10.2337/dc15-1095 26721810 PMC5884095

[7] Muschik D, Tetzlaff J, Lange K, Epping J, Eberhard S, Geyer S. Change in life expectancy with type 2 diabetes: a study using claims data from lower Saxony, Germany. Popul Health Metr. 2017; 15: 5 10.1186/s12963-017-0124-6 28193279 PMC5307777

[8] National diabetes register. Available from: https://usbale.org/wp-content/uploads/2018/03/Doklad_Diabetes_Reg_2017_.pdf.

[9] Borissova A-M, Shinkov A, Kovatcheva R, Vlahov J, Dakovska L, Todorov T. Changes in the Prevalence of diabetes Mellitus in Bulgaria (2006–2012). Clinical Medicine Insights: Endocrinology and Diabetes. 2015; 8: 41–45. 10.4137/CMED.S24742 26005363 PMC4431479

[10] Borissova A-M, Shinkov A, Vlahov I, Dakovska L, Blajeva E, Todorov T. Prevalence of Diabetes Mellitus and Prediabetes in Bulgaria Today. Endocrinologia Jornal. 2012; 4: 182–192 (Article in Bulgarian).

[11] World Health Statistics 2019: Monitoring health for the SDGs–World Health Organization. 2019; 1–132 Available from: https://www.who.int/gho/publications/world_health_statistics/2019/en/

[12] Jha P, MacLennan M, Chaloupka FJ, Yurekli A, Ramasundarahettige C, Palipudi K, et al Global Hazards of Tobacco and the Benefits of Smoking Cessation and Tobacco Taxes In: Gelband H, Jha P, Sankaranarayanan R, et al, editors. Cancer: Disease Control Priorities, Third Edition (Volume 3). Washington (DC): The International Bank for Reconstruction and Development / The World Bank; 2015 Chapter 10. Available from: https://www.ncbi.nlm.nih.gov/books/NBK343639/. 10.1596/978-1-4648-0349-9_ch10 26913345

[13] Rehm J, Shield KD. Alcohol and Mortality. Global Alcohol-Attributable Deaths From Cancer, Liver Cirrhosis, and Injury in 2010. Alcohol Res. 2014; 35 (2): 174–183. 10.35946/arcr.v35.2.07 PMC3908708 24881325

[14] US Bureau of Labor Statistics. National Census of Fatal Occupational injuries in 2015. 2016; 1–11. Available from: https://www.bls.gov/news.release/archives/cfoi_12162016.pdf

[15] Saeedi P, Petersohn I, Salpea P, Malanda B, Karuranga S, Unwin N, et al IDF Diabetes Atlas Committee. Global and regional diabetes prevalence estimates for 2019 and projections for 2030 and 2045: Results from the International Diabetes Federation Diabetes Atlas, 9th edition. Diabetes Res Clin Pract. 2019; 157: 107843 10.1016/j.diabres.2019.107843 31518657

[16] Krolewski A, Warram J, Christlieb A, Busick EJ, Kahn CR. The changing natural history of nephropathy in type I Diabetes. The American Journal of Medicine. 1985; 78 (5): 785–794. 10.1016/0002-9343(85)90284-0 3993659

[17] Holman RR, Paul SK, Bethel MA, Matthews DR, Neil HA. 10‑year follow‑up of intensive glucose control in type 2 diabetes. N Engl J Med. 2008; 359: 1577‑1589. 10.1056/NEJMoa0806470 18784090

[18] Loukine L, Waters C, Choi BC, Ellison J. Impact of diabetes mellitus on life expectancy and health adjusted life expectancy in Canada. Popul. Health Metr. 2012; 10 (1): 7 10.1186/1478-7954-10-7 22531113 PMC3787852

[19] Livingstone SJ, Levin D, Looker HC, Lindsay RS, Wild SH, Joss N, et al Estimated life expectancy in a Scottish cohort in type 1 diabetes, 2008–2010. JAMA. 2015; 313 (1): 37–44. 10.1001/jama.2014.16425 25562264 PMC4426486

[20] Lutgers HL, Gerrits EG, Sluiter WJ, Ubink-Veltmaat LJ, Landman GW, Links TP, et al Life expectancy in a large cohort of type 2 diabetes patients treated in primary care (ZODIAC-10). Plos One. 2009; 4 (8): e6817 10.1371/journal.pone.0006817 19714245 PMC2729379

[21] Hou L, Shaw JE, Wong E, Harding JL, Peeters A, Magliano DJ. Burden of diabetes in Australia: life expectancy and disability free life expectancy in adults with diabetes. Diabetologia. 2016; 59 (7): 1437–1445. 10.1007/s00125-016-3948-x 27075450

[22] Tachkov KT, Mitov KV, Mitkova ZE, Kamusheva MS, Dimitrova MJ, Petkova VB, et al Improved quality of diabetes control reduces complication costs in Bulgaria. Biotechnology & Biotechnological Equipment. 2019; 33(1): 814–820. 10.1080/13102818.2019.1604160

[23] Leal J, Gray AM, Clarke PM. Development of life expectancy tables for people with type 2 diabetes. European Heart Journal. 2009; 30 (7): 834–839. 10.1093/eurheartj/ehn567 19109355 PMC2663724

[24] Dale AC, Nilsen TI, Vatten L, Midthjell K, Wiseth R. Diabetes mellitus and risk of fatal ischemic hearth diseases by gender: 18 years follow up of 74914 individuals in the HUNT 1 study. Eur Heart Journal. 2007; 28 (23): 2924–2929. (10.1093/eurheartj/ehm447) 17947212

[25] Gu K, Cowie CC, Harris MI. Mortality in adult with and without diabetes in a national cohort of the U.S. population, 1971–1993. Diabetes care. 1998; 21 (7): 1138–1145. 10.2337/diacare.21.7.1138 9653609

[26] Roper NA, Bilous RW, Kelly WF, Unwin NC, Connolly VM. Excess mortality in a population with diabetes and the impact of material deprivation: longitudinal, population based study. BMJ. 2001; 322: 1389–1393. 10.1136/bmj.322.7299.1389 11397742 PMC32252

[27] Ostrowska J, Jakubczyk M, Niewada M, Lipka I, Petrova G, Tcharaktchiev D, et al Estimating the costs of diabetes-related cardiovascular complications in selected Central and Eastern European countries. Value in Health. 2017; 20: A476 10.1016/j.jval.2017.08.442

[28] Dyakova M, Shipovenska E, Dyakov P, Dimitrov P, Torbova S. Cardiovascular Risk assessment of Bulgarian Urban Population: Cross-sectional study. Croat Med J. 2008; 49 (6): 783–791. 10.3325/cmj.2008.49.783 19090603 PMC2621028

